# Is Zimbabwe ready to transition from anonymous unlinked sero-surveillance to using prevention of mother to child transmission of HIV (PMTCT) program data for HIV surveillance?: results of PMTCT utility study, 2012

**DOI:** 10.1186/s12879-016-1425-2

**Published:** 2016-02-29

**Authors:** E. Gonese, A. Mushavi, M. Mungati, M. Mhangara, J. Dzangare, O. Mugurungi, J. Dee, P. H. Kilmarx, G. Shambira, M. T. Tshimanga, J. Hargrove

**Affiliations:** Centers for Disease Control and Prevention, 38 Nelson Mandela Rd, Box 3088, Harare, Zimbabwe; Ministry of Health and Child Care Zimbabwe, Mukwati Building, Corner Livingstone/Fifth Street, Harare, Zimbabwe; Centers for Disease Control and Prevention, 1600 Clifton Road, Atlanta, GA 30329-4027 USA; National Institute of Health, 9000 Rockville Pike, Bethesda, MD 20892 USA; Department of Community Medicine, University of Zimbabwe, Parirenyatwa Hospital, Mazoe Street, Harare, Zimbabwe; South Africa Centre for Epidemiological Modelling and Analysis, Stellenbosch University, 19 Jonkershoek Road, Stellenbosch, 7602 South Africa

## Abstract

**Background:**

Prevention of mother-to-child transmission of HIV (PMTCT) programs collect socio-demographic and HIV testing information similar to that collected by unlinked anonymous testing sero-surveillance (UAT) in antenatal settings. Zimbabwe evaluated the utility of PMTCT data in replacing UAT.

**Methods:**

A UAT dataset was created by capturing socio-demographic, testing practices from the woman’s booking-card and testing remnant blood at a laboratory from 1 June to 30 September 2012. PMTCT data were collected retrospectively from ANC registers. UAT and PMTCT data were linked by bar-code labels that were temporarily affixed to the ANC register. A questionnaire was used to obtain facility-level data at 53 sites.

**Results:**

Pooled HIV prevalence was 15.8 % (95 % CI 15.3–16.4) among 17,349 women sampled by UAT, and 16.3 % (95 % CI 15.8 %–16.9 %) among 17,150 women in PMTCT datasets for 53 sites. Pooled national percent-positive agreement (PPA) was 91.2 %, and percent-negative agreement (PNA) was 98.7 % for 16,782 women with matched UAT and PMTCT data. Based on UAT methods, overall median prevalence was 12.9 % (Range 4.0 %–19.4 %) among acceptors and refusers of HIV test in PMTCT compared to 12.5 % ((Range 3.4 %–19.5 %) among acceptors in ANC registers. There were variations in prevalence by site.

**Conclusion:**

Although, there is no statistical difference between pooled HIV prevalence in UAT compared to PMTCT program, the overall PPA of 91.2 % and PNA of 98.7 % fall below World Health Organisation (WHO) benchmarks of 97.6 % and 99.6 % respectively. Zimbabwe will need to strengthen quality assurance (QA) of rapid HIV testing and data collection practices. Sites with good performance should be prioritised for transitioning.

**Electronic supplementary material:**

The online version of this article (doi:10.1186/s12879-016-1425-2) contains supplementary material, which is available to authorized users.

## Background

Over the past two decades, unlinked and anonymous HIV testing (UAT) for surveillance among pregnant women who routinely attend antenatal clinical (ANC) sentinel sites has provided valuable information about the burden of HIV and trends in HIV prevalence. Antenatal clinics provide an accessible cross-section of healthy, sexually active women in the general population, and HIV data from ANC surveillance are considered generally representative of the source community [1]. Epidemiologic information provided by ANC surveillance addresses key aspects of the “Know Your Epidemic” updated approach of second generation surveillance of HIV, understanding changes in the direction of the epidemic, understanding sub-national variations in an epidemic and identifying localized geographic areas with higher burdens of HIV [2]. This information is crucial for planning HIV control interventions and allocating required resources. ANC surveillance data also serve as one of the data sources used to construct mathematical models of HIV prevalence and incidence trends using Estimation and Projection Package (EPP)/Spectrum analysis tools developed by the Joint United Nations Programme on HIV/AIDS (UNAIDS) [3].

Globally, the scale-up of HIV care and treatment programs such as Prevention of Mother to Child Transmission (PMTCT) has highlighted the need to provide HIV test results in all settings, including surveillance, in order to increase access to care. Using the UAT methods, tests are conducted on left-over blood from routine testing at ANC (usually syphilis testing) where all personal identifiers are removed and therefore results are not returned to individuals. This approach has the potential to eliminate selection bias due to non-consent because, when testing is based on informed consent, individuals refusing testing may be at lower or higher risk of HIV infection than consenters [4–6]. There are, however, ethical concerns around using the UAT methods in conducting ANC HIV sentinel sero-surveillance (ANC HSS). This method does not obtain informed consent from pregnant women and therefore cannot return HIV testing results, or link HIV positive cases to HIV care, treatment, and prevention interventions, [7, 8]. In recent years there has been a growing global consensus around the imperative to routinely provide HIV test results to participants in HIV surveillance [9–11]. This has necessitated a change in surveillance data collection methods so that pregnant women can provide informed consent for accepting or declining an HIV test, receive the results in post-test counselling and accept referral into HIV care and treatment services during routine antenatal visits [2] 

PMTCT programs collect socio-demographic and HIV testing information similar to that collected by ANC HSS hence, many countries are considering the use of PMTCT program data to complement or replace UAT surveillance. High-quality PMTCT program data could provide an efficient and cost-effective alternative to UAT surveillance and enhance data use among health care workers.

The World Health Organisation (WHO) has provided guidelines to assess PMTCT programs data and quality in preparation of using PMTCT program data in replacing UAT surveillance [2]. Core elements to be assessed include: selection bias inherent in PMTCT-based HIV estimates, the coverage of PMTCT HIV testing services at sentinel sites, availability and completeness of a minimum set of surveillance variables in routine program records, accuracy of routine PMTCT HIV testing, and HIV testing quality assurance practices at ANC sites.

Since 2002, Zimbabwe has used biannual UAT at 19 sentinel sites to track trends in HIV prevalence [12]. All women accessing antenatal services in Zimbabwe are also offered PMTCT intervention using the opt-out of HIV testing strategy. As a result of the programmatic shift and scale up, Zimbabwe has considered transitioning to using PMTCT data to monitor trends in the HIV epidemic. The 2009 national ANC report highlighted shortfalls in data quality in the PMTCT registers. The report recommended revisions to the registers as well as focused health workers training on how to capture key indicators before transitioning to PMTCT program data for surveillance.

Following key interventions such as revisions to registers and training in record keeping training in rapid HIV testing and the scale up of the PMTCT program, we conducted a comprehensive assessment of the utility of PMTCT program data for ANC based surveillance in 2012. The overall objective was to assess the Zimbabwe Ministry of Health and Child Care (MOHCC) PMTCT programme’s readiness to use routine data for ANC based surveillance. We compared UAT surveillance HIV test results and prevalence estimates to those obtained from PMTCT programme in the same time period, assessed the completeness and quality of routine PMTCT programme data, as well as health facility testing and data recording practices.

## Methods

The assessment of the utility of using PMTCT program data for surveillance among pregnant women was implemented in three parts: (1) a comparison of HIV test results and prevalence estimates from UAT surveillance and routine PMTCT HIV testing during the same time period; (2) a retrospective data quality assessment of routine ANC records during the UAT surveillance period; (3) a health facility assessment to capture information on PMTCT HIV testing and data recording practices.

### Study sites and sample size

The sampling unit for this assessment was a sentinel site providing PMTCT HIV testing services. Of the initial target of 54 sentinel sites, 53 sites were included in the 2012 UAT surveillance and the PMTCT utility assessment. UAT forms from one site Karoi did not have the PMTCT results or samples for UAT testing and therefore a decision was made to exclude the site from analysis. Sites were chosen to represent all 8 provincial regions and 2 major cities in Zimbabwe.

The UAT surveillance sample size requirement was 340 each for all sentinel sites with the exception of large city health facilities, Kuwadzana, Nkulumane and St. Mary’s where the sample size requirement was 600. Detailed methods for sample size calculation for UAT are presented in the full MOHCC report [13].The PMTCT data quality assessment retrospectively collected data from all eligible women in the ANC register during the same period in which the UAT was conducted at all participating sites.

### Data collection tools and methods

#### UAT prospective surveillance

Between June and September 2012 the UAT survey prospectively enrolled women attending an ANC for the first time during the current pregnancy until the required samples size for the site was achieved. The attending nurse or midwife completed the UAT surveillance data collection form for each eligible woman by abstracting information from the mother’s booking card and ANC registers.

Firstly, all pregnant women were offered the routine antenatal clinic tests which included syphilis testing and blood grouping. Each woman who consented to routine ANC tests had 5 ml of blood drawn for routine ANC tests. The remnant blood sample was transferred into a separate anticoagulant tube for UAT surveillance. This blood was used to prepare Dried Blood Spots (DBS) on Whatman’s filter paper. Bar code labelled stickers were placed on the tube, DBS card and UAT surveillance form. UAT specimens were recorded on a tally sheet and transported to the National Microbiology Reference Laboratory (NMRL) via courier service for Syphilis confirmatory testing TPHA, and HIV testing using Enzyme Immuno-Assay (EIA) test kits. The initial test for HIV detection was conducted using AniLabsytems (AniLabsystems Ltd, Finland). All non-reactive result were reported negative as final result, while a reactive result was confirmed using *Enzygnost Anti HIV 1/2 plus ELISA* (Siemens Healthcare products, Munich Germany). Non-reactive samples by Enzygnost Anti HIV1/2 plus ELISA were further confirmed by Western Blot, New Lav Blot 1 (Biorad, France). Indeterminate Western blot results were further tested by HIV DNA, PCR (*ROCHE* Diagnostics, France)*.* The laboratory provided dataset with a final HIV result that was used in analysis. Results of NMRL testing were double entered into a database and sent, with UAT surveillance data collection forms, to the AIDS and TB Unit.

All pregnant women attending antenatal care were also offered HIV testing and counselling in ANC services and those who accepted received their results on the same visit day during post-test counseling. The HIV screening test was done using a Determine test kit. If the result was non- reactive, the woman was considered HIV-negative; a reactive test was confirmed using SD-Bioline and discrepant results were tested using INSTI™ HIV-1 Antibody test as a tiebreaker. All HIV results were recorded on the woman’s ANC booking card and in the ANC register. For this assessment, results of the rapid HIV test were entered from the register onto the UAT surveillance form that accompanied the UAT surveillance DBS, which was sent to the NMRL for UAT testing.

#### PMTCT retrospective data quality assessment

Health Information officers were assigned to retrospectively abstract PMTCT program data from the ANC register at the sentinel sites for the period June to September 2012, the same period in which the UAT surveillance was conducted at the 53 sites. During the UAT survey period, the site nurse affixed an extra bar code label in the ANC register for all women accepting PMTCT HIV testing and those who were not tested because they were either on ART or self –reported their status as HIV positive. The bar-code allowed us to conduct a head to head comparison of the UAT surveillance dataset and ANC register data. The key variables captured by the UAT surveillance form and also captured from the ANC registers included (1) demographic information (age, marital status, usual residence and educational level of client); (2) medical information (presence and history of sexually transmitted infections); (3) obstetric history (gravidity and parity) and (4) PMTCT-related information (HIV status at booking, offer and acceptance of PMTCT HIV testing at the current visit, current HIV test result, and whether the woman was currently on antiretroviral medication). The bar-codes in the ANC registers were removed from the registers when data abstraction and preliminary analysis of HIV test results from the NMRL was completed. The bar code allowed linkage of UAT results to PMTCT program data which had personal identifiable information. Realizing this, the MOHCC took action by returning to all sites and delinking the data by removing the bar codes affixed to the registers. This was reported to the Medical Research Council of Zimbabwe and permission was sought to follow up on the initially 14 women who were supposed to be HIV negative and on ART. Two women were later excluded from being followed up after data verification using PMTCT registers showed that the women were correctly recorded as HIV negative and that they were not on ART as reported on the UAT form.

#### Health facility assessment

The purpose of the facility assessment was to determine whether PMTCT HIV testing, data collection and record keeping were standard across sites in order to ensure availability of complete and valid PMTCT program data. At each of the 53 facilities we administered a structured questionnaire to one senior member of staff to obtain information on administrative ownership as defined by whether facility was owned by government, religious affiliation (mission) or municipal owned, services offered and HIV testing and referral practices. We collected qualitative information on patient flow, HIV and syphilis testing practices (including the managing and recording of women who are known to be HIV positive at booking). We also collected information on the availability and completeness of key data elements such as age, gravidity, parity and HIV status at booking. Although the target number of sites was initially 54, one site, UAT surveillance forms received from Karoi hospital did not have PMTCT results nor samples for UAT testing and was therefore excluded from further analysis.

Of the 53 sites, two facility assessments reports, for Chimhanda and Plumtree were misplaced and could not be traced between the facility and national office leaving 51 facilities in the health facility assessment analysis. The unavailability of this information did not affect the primary analysis of comparing HIV results from the PMTCT program and results from the ANC register.

### Data analysis

We used the UAT surveillance and PMTCT dataset from the ANC register to generate frequency tables, pooled site prevalence and individual analysis of HIV results using *STATA version 12*.

### Inclusion and exclusion for UAT and PMTCT datasets (Fig. 1)

**Fig. 1:**
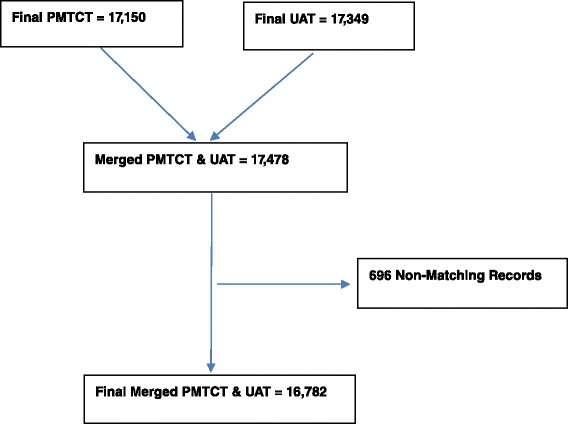
PMTCT program data and UAT surveillance data sets

A total of 17,349 women were considered for analysis in the by UAT surveillance and 17,150 women from PMTCT register records at 53sites. The two datasets were further cleaned and merged to provide a dataset with 17, 478 records, of which a total of 16,782 (96.0 %) where matched i.e. had both UAT and PMTCT program result. A total of 696 records were excluded because they did not appear either in the ANC or the UAT surveillance datasets. Sites which had high numbers of unmatched results in UAT surveillance included Morgenster (103) and Silveira (111) and for PMTCT Hauna (115) and Rusape (215). Although efforts were made to recover samples from Hauna and Rusape were not received at the NMRL and hence they were excluded from some analysis. This resulted in different denominators for the different analysis.

### Comparison of prevalence HIV estimates derived from program data and UAT surveillance

HIV prevalence estimates as recorded on the UAT surveillance form were compared for each site. HIV prevalence from program data was based on PMTCT HIV test results as recorded on the UAT surveillance data collection form by the site nurse. Women who were recorded as known HIV-positive at booking in ANC register data were considered HIV-positive for PMTCT program data Pooled site and national HIV estimates were calculated after excluding these missing results. Individual-level agreement of PMTCT and UAT surveillance HIV testing results was calculated among all women with both test results as recorded on the UAT surveillance form. These data were cross-tabulated to calculate positive percent agreement (PPA) (same calculation as sensitivity) and negative percent agreement (PNA) (the same calculation as specificity) of PMTCT and UAT surveillance HIV testing. This gives a measure of the proportion of individual cases giving results in UAT and PMTCT as either both positive or negative.

### Analysis of bias

Percent bias is defined by the positive or negative change of the HIV prevalence among women who did and did not accept PMTCT HIV testing (UAT) and those who did accept PMTCT HIV testing. We used data captured on the UAT surveillance form to analyse for selection bias inherent in program data due to non-uptake of PMTCT HIV testing. The HIV prevalence (according to UAT surveillance testing) among women who did, and did not, consent to PMTCT HIV testing (the population captured by current surveillance methods) was compared to the HIV prevalence among women who did consent to PMTCT HIV testing (the population that would have HIV test results available in a system of surveillance based on program data). Percent bias was calculated as the percent change (positive or negative) from the total HIV prevalence (among both PMTCT HIV testing accepters and non-acceptors) to the observed HIV prevalence (PMTCT HIV testing acceptors only). A total of 1, 176 (6.6 %) women who were known HIV-positive at booking (according to the ANC register) were excluded from this analysis as they would not be expected to have the opportunity to accept or decline an HIV test. Women with missing values for acceptance of PMTCT HIV testing on the UAT surveillance form were also excluded from the analysis.

### Analysis of data quality

We analysed the completeness and concordance of selected demographic variables of age, gravidity, parity, HIV status at booking, ART medication status at booking and PMTCT HIV result by comparing results recorded on the UAT surveillance form with the results in the ANC register data, using the bar-code label as a temporary matching link for the purposes of a head to head comparison of results. The analysis team did not have access to personal identifying information. The analysis team did not have access to personal identifying information and bar codes were removed from registers by site staff.

### Analysis of site assessment data

Quantitative results of the sites assessments were analyzed using simple descriptive analysis. We described the frequency of optimal and sub-optimal PMTCT HIV testing and data recording practices at surveillance sentinel sites. Of particular interest were non-standard practices, such as unavailability of onsite rapid HIV testing that could negatively affect the uptake of PMTCT HIV testing and the quality and completeness of program data. We used a site assessment tool which had yes/no response to elicit this information from onsite managers.

### Data validation process

Each site had an onsite supervisor who performed preliminary data quality and completeness checks. This cadre received further support from support and supervisory visits from higher levels. However final data checks were conducted at time of receipt by the National Surveillance Office and at point of data analysis. Forms were checked for completeness and consistency. Sites were notified of errors arising from their sites and requested to improve data quality. However, because of the constrained human resources during time of survey, this was not always observed, hence the high number of missing forms, incomplete data and general poor data quality.

### Ethical considerations

Approval to conduct the study was received from the Medical Research Council of Zimbabwe and a non-research determination was received from the Center for Global Health (CGH), Centers for Disease Control and Prevention (CDC) Atlanta.

We did not request consent to participate from the pregnant women. By definition, unlinked anonymous testing (UAT) surveillance does not inform women that they would be tested for surveillance, therefore we did not obtain individual consent from the women. However, because PMTCT is a priority intervention of the Government of Zimbabwe Ministry of Health and Child Care (MOHCC), all women are offered HIV testing and counselling in the context of PMTCT program. The bar code link was a temporary link which was removed and delinked soon after the survey to avoid tracing back to participants. Routinely site staff conducting the assessment are expected to observe the highest level of confidentiality as required by their professional codes of ethics and provision of HIV testing services in PMTCT. Additional safeguards were implemented through training of site staff and ensuring that all UAT surveillance data collection forms were stored in locked drawers at all sites and transferred to NMRL, where HIV test results were stored in a password protected database, which only the laboratory survey administrator could edit.

After data from the UAT surveillance form and the ANC register had been matched for individual records by the analysis team, the site staffs were requested to remove the survey bar-code label from the ANC registers, except in the case of 12 women who had discrepant results and required follow up. Ethical approval was received from MRCZ to follow up these women and retest to confirm status. The full report of this supplementary activity of following 12 women with discordant results in the 2012 ANC/PMTCT Assessment is available from the Ministry of Health and Child Care, Zimbabwe.

## Results

### Comparison of UAT and PMTCT program data

The results for 53 sentinel sites showed that, among 17,349 women sampled during the UAT surveillance period the pooled HIV prevalence was 15.8 % (95 % CI 15.3–16.4) with a range 7.37 % at Karanda to 25.61 % at Gwanda, Among the 17,150 women participating in PMTCT care compared to their information from the ANC register, the pooled HIV prevalence was 16.3 % (95 % CI 15.8–16.9) with a range of 8.28 % at St Alberts and Karanda and 30.60 % at Gwanda (Table 1). At 37 of the 53 sites (67.9 %) of the UAT surveillance result was slightly lower than the PMTCT program result.

**Table 1:** HIV prevalence estimates from UAT surveillance and PMTCT program data in ANC register

Site	HIV prevalence according to UAT surveillance testing (*n =* 17,349)	HIV prevalence according to program data^a^ (*n =* 17,150)	*p*-value	95 % C.I (Testing difference between HIV prevalence data according to UAT and PMTCT program data)
Banket	16.52	16.98	0.87	(−0.053; 0.062)
Beitbridge	22.36	22.46	0.97	(−0.063; 0.065)
Bindura	18.67	17.88	0.79	(−0.067; 0.051)
Binga	8.08	8.86	0.72	(−0.040; 0.051)
Chimhanda	9.57	10.54	0.68	(−0.037; 0.057)
Chinhoyi	14.16	14.11	0.99	(−0.053; 0.053)
Chiredzi	16.61	16.97	0.90	(−0.054; 0.061)
Chivi	12.04	13.72	0.52	(−0.035; 0.068)
Concession	15.62	17.05	0.62	(−0.043; 0.072)
Epworth	19.71	19.88	0.95	(−0.058; 0.062)
Filabusi	23.35	23.93	0.86	(−0.059; 0.071)
Glen View	10.21	9.31	0.70	(−0.054; 0.036)
Gokwe	11.34	12.75	0.59	(−0.037; 0.065)
Guruve	11.21	11.28	0.98	(−0.048; 0.049)
Gutu	15.88	16.77	0.76	(−0.047; 0.065)
Gwanda	25.61	30.6	0.16	(−0.019; 0.119)
Gweru	19.17	19.21	0.99	(−0.059; 0.060)
Hatcliffe	9.73	10.84	0.64	(−0.035; 0.057)
Hauna	10.31	11.32	0.74	(−0.048; 0.069)
Hwedza	17.26	17.31	0.99	(−0.057; 0.058)
Inyathi	20.67	23.51	0.38	(−0.036; 0.092)
Kadoma	14.98	16.39	0.63	(−0.043; 0.071)
Karanda	7.37	8.28	0.66	(−0.032; 0.050)
Kariba	14.58	15.09	0.85	(−0.049; 0.060)
Kuwadzana	11.69	11.66	0.99	(−0.037; 0.036)
Kwekwe	14.79	13.94	0.75	(−0.062; 0.045)
Mabvuku	12.36	16.31	0.36	(−0.039; 0.119)
Makumbe	19.33	19.41	0.98	(−0.061; 0.063)
Marondera	19.29	19.52	0.94	(−0.058; 0.062)
Masvingo	13.62	13.48	0.97	(−0.064; 0.061)
Murambinda	17.56	19.06	0.62	(−0.044; 0.074)
Murehwa	17.69	16.61	0.73	(−0.072; 0.051)
Musume	18.08	16.88	0.70	(−0.074; 0.050)
Mutambara	8.87	9.15	0.91	(−0.044; 0.050)
Mutoko	15.02	13.97	0.71	(−0.065; 0.044)
Mvuma	18.96	19.02	0.99	(−0.061; 0.062)
Ndanga	16.62	13.9	0.33	(−0.082; 0.027)
Neshuro	13.11	13.79	0.80	(−0.046; 0.059)
Nkayi	21.40	21.60	0.96	(−0.071; 0.075)
Nkulumane	17.11	18.01	0.69	(−0.035; 0.052)
Nyamandlovu	24.55	24.54	1.00	(−0.066; 0.066)
Nyanga	14.60	14.06	0.86	(−0.065; 0.054)
Plumtree	23.55	30.29	0.06	(0.002; 0.136)
Rusape	21.93	18.64	0.53	(−0.136; 0.071)
Sadza	14.75	14.94	0.95	(−0.052; 0.056)
Sakubva	14.03	14.45	0.88	(−0.049; 0.057)
Shamva	15.48	14.37	0.69	(−0.065; 0.043)
Shurugwi	16.20	16.56	0.90	(−0.054; 0.062)
Silveira	12.70	14.48	0.59	(−0.046; 0.081)
St Alberts	8.33	8.28	0.98	(−0.044; 0.043)
St Mary’s	11.17	10.17	0.58	(−0.045; 0.025)
Victoria Falls	23.6	25.39	0.59	(−0.048; 0.083)
Zvishavane	21.3	23.24	0.55	(−0.044; 0.083)
Total	15.86 (95 % CI 15.32–16.41)	16.30 (95 % CI 15.74–16.85)	0.27	(−0.003; 0.012)

^a^Includes women who received a positive PMTCT HIV test (as recorded on the UAT surveillance data collection form) and women who were known HIV positive at booking (as recorded in the ANC register)

Among 17,349 women sampled during the UAT surveillance period, 16,782 (96.0 %) had UAT surveillance and PMTCT HIV test results as recorded on the UAT surveillance data collection form. Individual level discrepancies were observed between the onsite PMTCT rapid HIV test results and ELISA tests results from the NMRL as recorded on the UAT surveillance form. Discrepant results were noted in 185 women who were HIV positive according to PMTCT HIV test results as recorded on the UAT surveillance data collection form but ELISA negative according to NMRL results. Similarly another 185 samples were rapid HIV test negative PMTCT HIV test results as recorded on the UAT surveillance data collection form but ELISA positive at NMRL. The pooled national PPA of PMTCT results to UAT surveillance was 91.2 %, and the pooled national PNA was 98.7 % (Table 2) for the 53 sites. Twenty-two sites had individual level PPA below 91 % and nine sites had a PNA below 98 %. These discrepant samples were retested using DNA PCR by another laboratory. Of the 185 samples which were initially positive in PMTCT only 89 remained positive and of the 185 initially negative samples only 173 were available for re-test and of these 117 (67.6 %) tested HIV positive. Following retest of the discrepant samples, the PPA of PMTCT data with UAT was 94.5 % and PNA was 99.5 %.

**Table 2:** Individual-level agreement of PMTCT HIV testing results^a^ and UAT surveillance (*n =* 16,782)

Site	Number of women^b^	Percent HIV positive according to UAT surveillance testing	^a^PMTCT+/UAT +	^a^PMTCT+/UAT -	^a^PMTCT-/UAT +	^a^PMTCT-/UAT -	Positive percent agreement	Negative percent agreement
Banket	317	16.7 %	49	4	3	260	94.2 %	98.5 %
Beitbridge	305	17.0 %	50	2	2	249	96.2 %	99.2 %
Bindura	324	16.7 %	47	7	4	264	92.2 %	97.4 %
Binga	303	4.3 %	12	1	2	287	85.7 %	99.7 %
Chimhanda	313	8.9 %	27	1	3	277	90.0 %	99.6 %
Chinhoyi	322	11.5 %	32	5	2	280	94.1 %	98.2 %
Chiredzi	329	15.8 %	47	5	4	260	92.2 %	98.1 %
Chivi	315	9.8 %	28	3	3	269	90.3 %	98.9 %
Concession	291	12.7 %	35	2	3	249	92.1 %	99.2 %
Epworth	336	19.6 %	61	5	5	265	92.4 %	98.1 %
Filabusi	301	18.3 %	50	5	3	243	94.3 %	98.0 %
Glen View	333	10.2 %	29	5	2	296	93.5 %	98.3 %
Gokwe	302	10.3 %	29	2	5	266	85.3 %	99.3 %
Guruve	321	8.7 %	26	2	4	283	86.7 %	99.3 %
Gutu	320	12.5 %	39	1	3	277	92.9 %	99.6 %
Gwanda	279	18.6 %	49	3	8	218	86.0 %	98.6 %
Gweru	304	13.8 %	37	5	2	260	94.9 %	98.1 %
Hatcliffe	318	9.1 %	29	0	0	288	100.0 %	100.0 %
Hauna	208	8.7 %	18	0	1	189	94.7 %	100.0 %
Hwedza	311	10.3 %	31	1	1	277	96.9 %	99.6 %
Inyathi	315	17.1 %	51	3	8	249	86.4 %	98.8 %
Kadoma	302	12.3 %	34	3	8	254	81.0 %	98.8 %
Karanda	313	3.5 %	11	0	2	300	84.6 %	100.0 %
Kariba	308	11.4 %	34	1	3	267	91.9 %	99.6 %
Kuwadzana	590	11.4 %	64	3	3	519	95.5 %	99.4 %
Kwekwe	325	12.6 %	38	3	1	283	97.4 %	99.0 %
Mabvuku	326	3.4 %	11	0	3	75	78.6 %	100.0 %
Makumbe	297	17.5 %	46	6	3	236	93.9 %	97.5 %
Marondera	317	15.1 %	47	1	2	267	95.9 %	99.6 %
Masvingo	309	10.0 %	27	4	3	275	90.0 %	98.6 %
Murambinda	289	9.3 %	25	2	2	260	92.6 %	99.2 %
Murehwa	285	16.5 %	42	5	1	237	97.7 %	97.9 %
Musume	310	13.2 %	38	3	3	212	92.7 %	98.6 %
Mutambara	286	7.3 %	20	1	2	252	90.9 %	99.6 %
Mutoko	318	14.8 %	42	5	0	271	100.0 %	98.2 %
Mvuma	290	13.4 %	34	5	5	241	87.2 %	98.0 %
Ndanga	317	12.9 %	29	12	3	272	90.6 %	95.8 %
Neshuro	316	11.4 %	32	4	4	268	88.9 %	98.5 %
Nkayi	225	13.3 %	25	5	0	193	100.0 %	97.5 %
Nkulumane	557	13.6 %	74	2	2	475	97.4 %	99.6 %
Nyamandlovu	304	19.4 %	46	13	10	233	82.1 %	94.7 %
Nyanga	249	12.9 %	26	6	3	213	89.7 %	97.3 %
Plumtree	278	16.2 %	43	2	15	217	74.1 %	99.1 %
Rusape	113	17.7 %	17	3	0	88	100.0 %	96.7 %
Sadza	319	11.9 %	36	2	4	277	90.0 %	99.3 %
Sakubva	340	14.1 %	46	2	4	284	92.0 %	99.3 %
Shamva	334	15.6 %	47	5	1	280	97.9 %	98.2 %
Shurugwi	296	12.8 %	30	8	9	233	76.9 %	96.7 %
Silveira	319	9.1 %	26	3	10	267	72.2 %	98.9 %
St Alberts	298	6.7 %	19	1	0	278	100.0 %	99.6 %
St Mary’s	600	11.2 %	59	8	2	531	96.7 %	98.5 %
Victoria Falls	282	14.2 %	39	1	2	240	95.1 %	99.6 %
Zvishavane	303	16.2 %	45	4	7	247	86.5 %	98.4 %
Total	16,782	12.6 %	1,928	185	185	14,051	91.2 %	98.7 %

^a^PMTCT HIV testing results as recorded on the UAT surveillance data collection form

^b^Excludes women with a missing PMTCT HIV test result on the UAT surveillance data collection form

Using results on the UAT surveillance form, the overall uptake of PMTCT at the 53 sites was 95.2 %. The overall percent bias introduced by PMTCT was −2.6 % (Table 3). At least 1,176 women had an HIV positive result prior to booking with current pregnancy. Among these women, at least 705 (60 %) were already receiving ART, but this did not include the 12 women whose PMTCT program results and NMRL ELISA result were discordant according to UAT form. The concordance rate for HIV results in UAT and those in the PMTCT register for women already on ART was 95.3 %. The overall median HIV prevalence (based on UAT surveillance testing) among all women, including those who either accepted or refused a PMTCT HIV test, was 12.9 % (Range 4.0 %–19.4 %). In comparison, the median HIV prevalence among women who did accept a PMTCT HIV test was 12.5 % (Range 3.4 %–19.5 %) resulting in an overall percent bias of (−2.6 %). Eighteen sites had percent bias below (−2.6 %), while fifteen sites reported a bias of 0.0 %. Table 3

**Table 3:** Selection bias in non-acceptors^a^ of PMTCT HIV testing^b^

Site	Number and HIV prevalence according to surveillance testing among women who did and did not accept PMTCT HIV testing (*n =* 16,719)		Number and HIV prevalence according to surveillance testing among women who accepted PMTCT HIV testing (*n =* 16,597)		Percent bias^c^
Banket	332	16.6	330	16.1	−3.1 %
Beitbridge	303	18.5	294	16.3	−11.6 %
Bindura	321	16.5	320	16.3	−1.6 %
Binga	316	5.4	304	4.3	−20.4 %
Chimhanda	319	9.4	315	9.2	−2.0 %
Chinhoyi	319	11.9	318	11.6	−2.3 %
Chiredzi	313	16.3	313	16.3	0.0 %
Chivi	310	9.7	307	9.8	0.9 %
Concession	319	13.2	315	12.4	−6.0 %
Epworth	339	19.5	339	19.5	0.0 %
Filabusi	308	19.2	303	17.8	−7.0 %
Glen View	333	10.2	333	10.2	0.0 %
Gokwe	325	9.9	325	9.9	0.0 %
Guruve	324	9.6	323	9.6	0.3 %
Gutu	320	11.9	319	11.6	−2.4 %
Gwanda	282	18.8	277	18.1	−3.9 %
Gweru	306	13.1	304	12.5	−4.4 %
Hatcliffe	330	8.5	329	8.2	−3.2 %
Hauna	215	8.4	214	7.9	−5.1 %
Hwedza	308	10.7	305	10.5	−2.1 %
Inyathi	306	16.7	303	16.5	−1.0 %
Kadoma	320	13.1	318	12.6	−4.2 %
Karanda	326	4.0	324	3.4	−14.8 %
Kariba	319	11.9	318	11.6	−2.3 %
Kuwadzana	595	11.6	595	11.6	0.0 %
Kwekwe	330	13.3	324	12.7	−5.1 %
Mabvuku	89	12.4	89	12.4	0.0 %
Makumbe	317	18.3	313	17.9	−2.2 %
Marondera	319	15.1	314	14.3	−4.8 %
Masvingo	317	11.0	316	10.8	−2.5 %
Murambinda	303	9.9	300	9.3	−5.8 %
Murehwa	290	15.5	290	15.5	0.0 %
Musume	272	16.9	272	16.9	0.0 %
Mutambara	285	7.4	285	7.4	0.0 %
Mutoko	330	14.2	330	14.2	0.0 %
Mvuma	300	13.3	298	12.8	−4.4 %
Ndanga	321	13.4	318	12.6	−6.1 %
Neshuro	315	11.1	315	11.1	0.0 %
Nkayi	237	17.3	219	13.7	−20.8 %
Nkulumane	551	13.6	551	13.6	0.0 %
Nyamandlovu	309	19.4	306	19.3	−0.7 %
Nyanga	262	12.2	261	11.9	−2.7 %
Plumtree	282	16.0	281	16.0	0.3 %
Rusape	109	18.4	108	18.5	0.9 %
Sadza	316	11.4	316	11.4	0.0 %
Sakubva	326	12.6	326	12.6	0.0 %
Shamva	334	15.6	331	15.4	−1.0 %
Shurugwi	286	12.9	284	12.7	−2.0 %
Silveira	313	9.3	312	9.0	−3.2 %
St Alberts	299	6.7	297	6.7	0.6 %
St Mary’s	599	11.2	599	11.2	0.0 %
Victoria Falls	291	14.1	289	13.8	−1.8 %
Zvishavane	309	15.9	308	15.6	−1.8 %
Total	16,719	12.9 %	16,597	12.5 %	−2.6 %

^a^Acceptance of PMTCT HIV testing as recorded on the UAT surveillance data collection form

^b^Excluding those who were known HIV-positive at booking as recorded in the ANC register

^c^Percent bias is defined as the percent change, positive or negative, from the HIV prevalence among women who did and did not accept PMTCT HIV testing to the HIV prevalence among women who accepted PMTCT HIV testing

Among the 16,782 women sampled from the ANC register during the same time period as the UAT surveillance period, percent completeness of data was as follows: 99.9 % for age, 99.4 % for gravidity, 98.9 % for parity, 86.9 % for HIV status at booking, 96.6 % for already on ART, and 98.5 % for PMTCT HIV test result.

An analysis of recording practices showed that, among HIV positive women who came to the clinic, 99.0 % were consistently recorded in the ANC register as HIV positive.

Site assessment data was completed for 51 of the 53 sites (Table 4). All of these 51 sites offered onsite rapid HIV testing in PMTCT sites. There were variations in HIV testing and recording of previous HIV test history. At least 11.8 % of sites offered an HIV test to women who came to the site already knowing their status. Among 43 sites (84.3 %) that did not offer a re-test to a woman who came to the clinic knowing her result, 6 sites (14.0 %) did not record any result. Of the 51 sites, 9 sites (17.7 %) of the sites did not offer routine syphilis tests to pregnant women. The assessment reported stock-out of rapid HIV test kits at a third of the sites in the year prior to this survey. Health staff at some facilities reported that the national office had changed the rapid testing algorithm from using Determine as the screening test kit to First Response. This change was not communicated to all sites. Following inadequate supplies of the kit and challenges in interpreting the results, sites reverted back to using Determinerapid HIV test kit.

**Table 4:** Characteristics of sentinel surveillance sites as collected on the site assessment questionnaire (*n =* 51)

Characteristic	Response	N (%)
Setting	Urban	25 (49.0)
	Rural	26 (51.0)
Ownership	Government	33 (64.7)
	Mission	8 (15.7)
	City council	10 (19.6)
	Private clinic	0 (0.0)
	Other	0 (0.0)
Onsite Rapid testing	Yes	51 (100.0)
	No	0 (0.0)
PMTCT testing approach	Opt out	47 (92.2)
	Opt in	3 (5.9)
	Missing	1 (1.9)
HIV test kits stock out in 2011	Yes	17 (33.3)
	No	33 (64.7)
Among sites reporting test kits stock out in 2011, the number of distinct instances of stock out	1–2	14 (100.0)
	Missing	3 (17.6)
Syphilis testing done on site	Yes	42 (82.4)
	No	9 (17.7)
ANC register is a longitudinal register	Yes	44 (86.3)
	No	5 (9.8)
	Missing	2 (3.9)
Women who are known HIV-positive at booking are still given an HIV test for PMTCT	Yes	6 (11.8)
	No	43 (84.3)
	Missing	2 (3.9)
Among those sites that do not test a women who is known HIV-positive at booking, what is recorded in the HIV test result field	Positive	18 (41.9)
	Known positive/on ARV	15 (34.9)
	Nothing recorded	6 (14.0)
	Other	4 (9.3)
Information is recorded to indicate that a pregnant woman already knows she is HIV positive	Yes	47 (92.2)
	No	2 (3.9)
	Missing	2 (3.9)
Among sites that do record the status of previously known positive, in what field is the information recorded	HIV status before booking	16 (34.0)
	Already on ART	2 (4.3)
	HIV test result in ANC	11 (23.4)
	Notes/comments	7 (14.9)
	Other	1 (2.1)
	Missing	9 (19.1)
What is recorded for “HIV test result” is a woman refuses an HIV test	Not applicable	1 (1.96)
	Refused	31 (60.8)
	Nothing recorded	2 (3.9)
	Other	17 (33.3)

## Discussion

There was no statistically significant difference between the pooled national HIV prevalence estimate obtained from UAT surveillance period, 15.8 % (95 % CI 15.3;16.4) compared to PMTCT programme data estimate of 16.3 % (95 % CI 15.8;16.9) during the same time period in Zimbabwe in the 2012 assessment. Additionally, there were no statistically significant variations across the sites for pooled HIV prevalence and this is consistent with studies elsewhere [14]. However, the overall PPA of 91.2 % and PNA of 98.7 % suggest that additional support for quality assurance of HIV testing is required before Zimbabwe can transition to the use of PMTCT programme data as a replacement for UAT surveillance. Sentinel surveillance sites with low score on the site assessment, poor PMTCT/NMRL testing concordance, or poor routine data quality will be targeted for support, but that all sites should benefit from measures to strengthen the quality of routine data, routine testing and QA for onsite rapid HIV testing including stock management and standardisation of data entry.

This assessment found a PMTCT uptake of 95.2 % and overall selection bias in PMTCT program data of −2.6 %. This finding means that non acceptance for an HIV test among pregnant women did not introduce a significant bias in the HIV prevalence estimates obtained from this survey. When uptake of PMTCT testing is high, the percent bias is lower and countries are in a favourable position for transitioning if other critical criteria are met. This finding is consistent with recent results from other countries with high rates of uptake of PMTCT HIV testing among ANC attendees [4–6]. Sites that reported no difference between UAT surveillance and PMTCT program data may signify high awareness of the benefits of PMTCT resulting in clients choosing to accept an HIV test rather than to opt-out.

The overall 91.2 % PPA and 98.7 % PNA between UAT and PMTC program data are a little lower than desirable for using PMTCT data for surveillance. Although these are not strict targets, the WHO, CDC and other health partners consider a PPA of 97.6 % and PNA of 99.7 % as desirable benchmarks for transitioning in countries with HIV prevalence among pregnant women of between 9 % and 16 % [2]. Studies conducted in Kenya and Mozambique on the agreements between routine HIV rapid testing and ELISA-based surveillance found substantial disagreement between the two algorithms, with PPA of PMTCT HIV testing and surveillance HIV testing at 88.5 % and 75.9 % for Mozambique and Kenya, respectively [4–6]. These discrepancies could potentially be explained by the higher sensitivity of ELISA test kits. The WHO has provided guidance to use rapid HIV test kits in serial and to confirm results using the Western Blot instead of using ELISA as this does not eliminate false positives [15]. The improvements of the PPA to 94.5 % and PNA to 99.5 % in this evaluation suggest the need to ensure that onsite algorithms include both highly sensitive and specific rapid test kits in order to improve accuracy of results. It is possible that test results are affected by antiretroviral use. The effect of low antibody as a result of antiretroviral therapy on the accuracy of rapid HIV testing is currently not documented. In Zimbabwe, variations in PPA and PNA resulted in some sites having large differences between HIV estimates based on UAT surveillance (NMRL ELISA test) and those from routine PMTCT rapid HIV testing, as observed at Beitbridge (−11.6 %), Binga (−20.4 %), Karanda (−14.8 %) and Nkayi (−20.8 %). These results show that UAT is proportionally lower than PMTCT prevalence estimates. Assuming that the UAT testing result is accurate, a PPA of 91.2 % means that approximately 9 out of every 100 HIV positive women receive an HIV negative result, and a PNA of 98.7 % translates to 13 out of every 1,000 HIV negative women receiving an HIV positive test result on the first visit. For women receiving a false negative result, it is crucial for health authorities to put in place follow-up testing policies that would allow the individuals to receive the correct result in future testing and have access to care. In the case of false positive results, it is crucial to have a testing algorithm which could include a follow on DNA-PCR and viral load tests in order to avoid the negative impact of putting HIV negative women on life-long treatment especially in the era of option B+. When this assessment was implemented, Zimbabwe was still working on revising testing algorithm to take into account the need for viral load testing. Furthermore, 22 of the 53 sites had individual level PPA below 91 % and 9 sites had a PNA below 98 %. This study highlighted the need for strengthening quality assurance for onsite rapid HIV tests at the individual sites. The National PMTCT program could consider a phased approach in transitioning to use of PMTCT programme data for sites with relatively high performance indicators.

The completeness of PMTCT data in ANC registers was high for most key variables except for the women’s reported HIV status at clinic entry. This was a significant improvement from the completeness of ANC registers reported in 2009 [12]. Previous assessments of the quality of routinely collected ANC data for use in surveillance have shown mixed results. Studies since 2000 in Botswana, Kenya and Uganda reported significant data quality limitations in ANC surveillance sites, including the lack of key surveillance variables (such as HIV status at booking) in ANC registers [10, 11, 16– 18]. However, a Botswana study conducted in 2009 found a high level of completeness of routine data in ANC surveillance sites [19].

The availability and high uptake of counselling and rapid HIV testing in PMTCT setting are key in the transitioning from UAT surveillance to using PMTCT program data. The assessment conducted in Zimbabwe in 2012 showed that while all sites offer onsite rapid HIV testing; there are some elements that still require strengthening. The stock out of rapid HIV test kits reported by at least 33.3 % of facilities in the last year point to the need for strengthening supply chain management. At least 17.7 % of sites also reported unavailability of routine syphilis test therefore showing absence of ancillary tests in care and treatment settings. A closely related problem is lack of quality assurance at facilities and that sites may alter testing algorithms, leaving them without either screening or confirmatory kits. Poor supply chain management also results in biased results arising from periods when test kits are available or unavailable [2].

### Limitations

Both UAT surveillance and PMTCT register datasets were characterized by missing or excluded records (Additional file 1: Table S1 and Additional file 2: Table S2). Although HIV test result in UAT surveillance was used as a reference point, it is conceivable that disagreement between UAT and PMTCT testing could have potential for false positives from using an EIA based algorithm.

## Conclusions

There was no statistically significant difference between pooled site level HIV prevalence from PMTCT data in the ANC register and UAT surveillance. However, the overall positive and negative percent agreements that were slightly lower than the WHO benchmark suggest that Zimbabwe should continue to reinforce the quality of onsite HIV testing and program data collection in preparation for using PMTCT data for surveillance purposes. However, sites that show good performance indicators should be prioritised for transitioning. This assessment highlights the need for Zimbabwe to strengthen the standardised rapid HIV testing algorithm, forecasting and quantification of the requirement for rapid HIV and syphilis test kits, and to put in place onsite quality assurance of rapid HIV testing and record keeping practices in order to transition to use PMTCT data for surveillance.

## Additional files

Additional file 1: Table S1. Merged Data Set. (PDF 26 kb) Additional file 2: Table S2. Missing samples. (DOC 117 kb)

## Abbreviations

AIDS: acquired immunodeficiency syndrome
ANC: antenatal clinic
ANC HSS: antenatal clinic hiv sero-surveillance
ART: antiretroviral therapy
CDC: United States Centers for disease control and prevention
CGH: center for global health
DBS: dried blood spot
DHS: demography and health survey
DQA: data quality assessment
HIV: human immunodeficiency virus
NMRL: national microbiology reference laboratory
PEPFAR: the president’s emergency plan for aids relief
PITC: provider initiated testing and counseling
PMTCT: prevention of mother-to-child transmission
PNA: percent negative agreement
PPA: percent positive agreement
QA: quality assurance
UAT: unlinked anonymous testing
UNAIDS: joint United nations programme on hiv/aids
WHO: world health organization
ZDHS: Zimbabwe demographic and health survey
