# Supplementary material for: Is Zimbabwe ready to transition from anonymous unlinked sero-surveillance to using prevention of mother to child transmission of HIV (PMTCT) program data for HIV surveillance?: results of PMTCT utility study, 2012
Source: BMC Infect Dis. 2016 Feb 29;16:97. doi: 10.1186/s12879-016-1425-2 (PMC4770682; doi:10.1186/s12879-016-1425-2)
Supplement: Additional file 1: Table S1. — Merged Data Set. (PDF 26 kb) [file 12879_2016_1425_MOESM1_ESM.pdf]

12832

# Ministry of Health & Child Welfare
# Antenatal Clinic Surveillance 2012

**1. Survey Number Sticker (big sticker on top)**

**INSTRUCTIONS**

Please fill in circles like this: ●

NOT like this: ∅ ⊗ ✓

**2.Age**

0 ⓪ ⓪
1 ① ①
2 ② ②
3 ③ ③
4 ④ ④
5 ⑤ ⑤
6 ⑥ ⑥
7 ⑦ ⑦
8 ⑧ ⑧
9 ⑨ ⑨

**3. Place of Usual Residence**

- ○ Rural
- ○ Mine
- ○ Growth Point
- ○ Farm
- ○ Town or City
- ○ Resettlement

**4. Type of Occupation**

- ○ Student
- ○ Housewife
- ○ Unemployed
- ○ Employed (Formal)
- ○ Employed (Informal)

**5. Partner's Type of Occupation**

- ○ Student
- ○ Unemployed
- ○ Employed (Formal)
- ○ Employed (Informal)
- ○ N/A

**6. Partner's Place of Usual Residence**

- ○ Rural
- ○ Farm
- ○ Growth Point
- ○ Resettlement
- ○ Town or City
- ○ Not known
- ○ Mine
- ○ N/A

**7. Marital Status**

- ○ Married
- ○ Separated
- ○ Single
- ○ Widowed
- ○ Divorced

**8. Partner's Educational Level**

- ○ None
- ○ Primary
- ○ Secondary (Junior)
- ○ Secondary (O' Level)
- ○ Secondary (A' Level)
- ○ Tertiary
- ○ N/A

**9. Educational Level**

- ○ None
- ○ Primary
- ○ Secondary (Junior Certificate)
- ○ Secondary (O' Level)
- ○ Secondary (A' Level)
- ○ Tertiary

**10. Genital Ulcer History**

| | Y | N |
|---|---|---|
| a) History of GUD within the past year? | ○ | ○ |
| b) Presence of GUD on examination | ○ | ○ |

**11. HIV Test History**

a) Prior to this visit, have you ever been offered an HIV test? ○ Yes ○ No

b)Was this HIV test accepted? ○ Yes ○ No ○ N/A

c) Did you receive results during post test counseling? ○ Yes ○ No ○ N/A

d) Prior to this visit did you ever take ARVs? ○ Yes ○ No ○ N/A

**12. Currently on ARV**

Y ○ N ○

**13. PMTCT HIV Test - Current Visit**

a) Was an HIV test offered? ○ Yes ○ No

b)Was this test accepted? ○ Yes ○ No ○ N/A

c) What was the HIV test result? ○ Positive ○ Negative ○ N/A

12832

## 14. Obstetric Information

| | 0 | 1 | 2 | 3 | 4 | 5 | 6 | 7 | 8 | 9 |
|---|---|---|---|---|---|---|---|---|---|---|
| a) Gravida | 0 | 1 | 2 | 3 | 4 | 5 | 6 | 7 | 8 | 9 |
| b) No. of Children Alive | 0 | 1 | 2 | 3 | 4 | 5 | 6 | 7 | 8 | 9 |
| c) Still Births | 0 | 1 | 2 | 3 | 4 | 5 | 6 | 7 | 8 | 9 |
| d) Abortions | 0 | 1 | 2 | 3 | 4 | 5 | 6 | 7 | 8 | 9 |
| e) No.of Children dead before 1st birthday | 0 | 1 | 2 | 3 | 4 | 5 | 6 | 7 | 8 | 9 |
| f) No. of children dead between 1st and 5th birthday | 0 | 1 | 2 | 3 | 4 | 5 | 6 | 7 | 8 | 9 |
| g) Parity | 0 | 1 | 2 | 3 | 4 | 5 | 6 | 7 | 8 | 9 |

## 15. Lab Results

| | DD | MM | YY |
|---|---|---|---|
| Collected | __ / | __ / | __ |
| Tested | __ / | __ / | __ |

| | + | - |
|---|---|---|
| HIV | ○ | ○ |
| RPR | ○ | ○ |
| TPHA | ○ | ○ |
