# Supplementary material for: Is Zimbabwe ready to transition from anonymous unlinked sero-surveillance to using prevention of mother to child transmission of HIV (PMTCT) program data for HIV surveillance?: results of PMTCT utility study, 2012
Source: BMC Infect Dis. 2016 Feb 29;16:97. doi: 10.1186/s12879-016-1425-2 (PMC4770682; doi:10.1186/s12879-016-1425-2)
Supplement: Additional file 2: Table S2. — Missing samples. (DOC 117 kb) [file 12879_2016_1425_MOESM2_ESM.doc]

**Supplementary Form 2: ANC/PMTCT Data Quality Assessment: Site Assessment Form**

INSTRUCTIONS: Please complete this form with the staff member who provides PMTCT services at the site. Provide the following information to the interviewee: “Today we would like to ask you some questions to describe this clinic and the way that this clinic collects PMTCT data. We are not here to assess your site’s performance, but rather to learn about the process of collecting information in PMTCT programs. This form should take approximately 30 minutes to complete.”

| 1. **Site Information** | | | | | | | | | | | | | | |
| --- | --- | --- | --- | --- | --- | --- | --- | --- | --- | --- | --- | --- | --- | --- |
| INSTRUCTIONS: The first section collects basic information about the site. | | | | | | | | | | | | | | |
| **1. Today’s date** | | | _ _ / _ _ / _ _ _ _ (m m / d d / y y y y ) | | | | | | | | | | | |
| **2. Interviewer’s name** | | |  | | | | | | | | | | | |
| **3. Health Facility Name** | | |  | | | | | | | | | | | |
| **4. County** | | |  | | | | | | | | | | | |
| **6. Setting** | | | □ Urban □ Rural □ | | | | | | | | | | | |
| **7. Facility operator** | | | □ Government □ Mission □ City Council □ Private clinic  □ NGO ⇨ Name of NGO ___________ | | | | | | | | | | | |
| **8. Average number of women enrolling in ANC for a new pregnancy each month** | | | _ __ women | | | | | | | | | | | |
| 1. **PMTCT Program Information** | | | | | | | | | | | | | | |
| INSTRUCTIONS: The second section collects information about HIV testing at the site and off-site. Note: explain to the interviewee that “at this site” means “in the building or compound that contains ANC and PMTCT services.” | | | | | | | | | | | | | | |
| **9. PMTCT program at site since** | | | _ _ / _ _ _ _ (m m / y y y y) | | | | | | | | | | | |
| **10. PMTCT testing approach** | | | □ Opt-in/ opt -out □ Mandatory □ VCT | | | | | | | | | | | |
| **11. Is HIV rapid testing done at this site?** | | | □ No ⇨ If No, go to next question  □ Yes ⇨ if Yes, skip to Question **18** | | | | | | | | | | | |
| **12. Where is off-site HIV testing done?** | | | □ Off-site laboratory  □ VCT site | | | | | | | □ Care and treatment center  □ Other _____________ | | | | |
| **13. What kind of test is used for off-site HIV testing?** | | | □ Rapid test specify name  □ Enzyme immunoassay specify name | | | | | | | | | | | |
| **14. What kind of sample is taken for the off-site HIV testing?** | | | □ Whole blood  □ Serum | | | | | | □ Plasma  □ Dried blood spot | | | | | |
| **15. When does a mother do her off-site HIV testing?** | | | □ Always the same day a mother is referred for testing  □ Sometimes the same day a mother is referred for testing  □ Never the same day a mother is referred for testing | | | | | | | | | | | |
| **16. How are off-site HIV test results physically returned to the PMTCT site?** | | | □ Returned by the testing site (lab, VCT site, etc)  □ Returned by the mother | | | | | | | | | | | |
| **17. When are off-site HIV test results physically returned to the PMTCT site?** | | □ Always the same day a mother is referred for testing  □ Sometimes the same day a mother is referred for testing  □ Never the same day a mother is referred for testing | | | | | | | | | | ⇨  ⇨  ⇨ | | Skip to  Question **20** |
| **18. PMTCT HIV testing algorithm for on-site rapid testing** | | | **18a.** Screening assay: | | | | | | | | | | | |
|  |  |  | **18b.** Confirmatory assay: | | | | | | | | | | | |
|  |  |  | **18c.** Tie breaker assay: | | | | | | | | | | | |
| **19. In [2011], was there ever a time when HIV test kits were unavailable due to stock outs?** | | | □ No  □ Yes ⇨ | | | | **19a.** If yes to 19, how many distinct instances of stock out were there in [YEAR]?  □ 1-2 □ 3-4 □ 5 or more | | | | | | | |
| **20a. In [2011], were women responsible for any out-of-pocket costs for any part of ANC services?**  **20b. In [2011], were women responsible for any out-of-pocket costs for any part of PMTCT services?** | | | □ No  □ Yes ⇨  □ No  □ Yes ⇨ | | | **20a.** Cost incurred for what? _______________  **20b.** Cost incurred for what? _______________ | | | | | | | | |
| **21. Is syphilis testing done at this site?** | | | □ No ⇨ If No, go to next question  □ Yes ⇨ if Yes, skip to Section **C** | | | | | | | | | | | |
| **22. Where is off-site syphilis testing done?** | | | □ Off-site laboratory  □ VCT site | | | | | | | | □ Care and treatment center  □ Other _____________ | | | |
| **23. When does a mother do her off-site syphilis testing?** | | | □ Always the same day a mother is referred for testing  □ Sometimes the same day a mother is referred for testing  □ Never the same day a mother is referred for testing | | | | | | | | | | | |
| **24. How are off-site syphilis test results physically returned to the PMTCT site?** | | | □ Returned by the testing site (lab, VCT site, etc)  □ Returned by the mother | | | | | | | | | | | |
| **25a. When are off-site syphilis test results physically returned to the PMTCT site?** | | | □ Always the same day a mother is referred for testing  □ Sometimes the same day a mother is referred for testing  □ Never the same day a mother is referred for testing | | | | | | | | | | | |
| **25b. In [2011], was there ever a time when syphilis test kits were unavailable due to stock outs?** | | | □ No  □ Yes ⇨ **25c.** If yes to 25b, how many distinct instances of stock out were there in [YEAR]?  □ 1-2 □ 3-4 □ 5 or more | | | | | | | | | | | |
| 1. **Patient Data Recording in the ANC and HIV Testing Registers** | | | | | | | | | | | | | | |
| INSTRUCTIONS: This section asks about the ANC register and the HIV testing register, what variables are routinely recorded in each register and when these variables are recorded. First, this section asks about variables in the ANC register, then it asks about variables in the HIV testing register (if separate from the ANC register). | | | | | | | | | | | | | | |
| **Questions 26 to 36 concern the ANC Register check consecutive 100 records** | | | | | | | | | | | | | | |
| **Variable** | **A. Recorded?** | | **B. When is variable first recorded?** | | | | | | | | | | | |
| **26. ANC number** | □ Recorded  □ Not recorded ⇨ Skip | | □ Always 1st visit  □ Always 2nd visit | | | | | □ Usually 1st visit, occasionally 2nd visit  □ Sometimes 1st visit, sometimes 2nd visit | | | | | | |
| **27. Date of visit** | □ Recorded  □ Not recorded ⇨ Skip | | □ Always 1st visit  □ Always 2nd visit | | | | | □ Usually 1st visit, occasionally 2nd visit  □ Sometimes 1st visit, sometimes 2nd visit | | | | | | |
| **28. Age** | □ Recorded  □ Not recorded ⇨ Skip | | □ Always 1st visit  □ Always 2nd visit | | | | | □ Usually 1st visit, occasionally 2nd visit  □ Sometimes 1st visit, sometimes 2nd visit | | | | | | |
| **29. Parity** | □ Recorded  □ Not recorded ⇨ Skip | | □ Always 1st visit  □ Always 2nd visit | | | | | □ Usually 1st visit, occasionally 2nd visit  □ Sometimes 1st visit, sometimes 2nd visit | | | | | | |
| **30. Gravidity** | □ Recorded  □ Not recorded ⇨ Skip | | □ Always 1st visit  □ Always 2nd visit | | | | | □ Usually 1st visit, occasionally 2nd visit  □ Sometimes 1st visit, sometimes 2nd visit | | | | | | |
| **31. Residence** | □ Recorded  □ Not recorded ⇨ Skip | | □ Always 1st visit  □ Always 2nd visit | | | | | □ Usually 1st visit, occasionally 2nd visit  □ Sometimes 1st visit, sometimes 2nd visit | | | | | | |
| **32. Occupation** | □ Recorded  □ Not recorded ⇨ Skip | | □ Always 1st visit  □ Always 2nd visit | | | | | □ Usually 1st visit, occasionally 2nd visit  □ Sometimes 1st visit, sometimes 2nd visit | | | | | | |
| **33. HIV test date** | □ Recorded  □ Not recorded ⇨ Skip | | □ Always 1st visit  □ Always 2nd visit | | | | | □ Usually 1st visit, occasionally 2nd visit  □ Sometimes 1st visit, sometimes 2nd visit | | | | | | |
| **34. HIV test result** | □ Recorded  □ Not recorded ⇨ Skip | | □ Always 1st visit  □ Always 2nd visit | | | | | □ Usually 1st visit, occasionally 2nd visit  □ Sometimes 1st visit, sometimes 2nd visit | | | | | | |
| **35. Syphilis test result** | □ Recorded  □ Not recorded ⇨ Skip | | □ Always 1st visit  □ Always 2nd visit  □ On availability of reagents | | | | | □ Usually 1st visit, occasionally 2nd visit  □ Sometimes 1st visit, sometimes 2nd visit | | | | | | |
| **36. On ART** | □ Recorded  □ Not recorded ⇨ Skip | | □ Always 1st visit  □ Always 2nd visit | | | | | □ Usually 1st visit, occasionally 2nd visit  □ Sometimes 1st visit, sometimes 2nd visit | | | | | | |
| **Questions 37 to 48 concern the Laboratory Testing Register** | | | | | | | | | | | | | | |
| **37. Are the HIV testing log and the ANC log both contained in one physical register?** | | | □ No ⇨ If No, go to next question  □ Yes ⇨ If Yes, skip to Section **D** | | | | | | | | | | | |
| **38. ANC number** | □ Recorded  □ Not recorded ⇨ Skip | | □ Always 1st visit  □ Always 2nd visit | | | | | □ Usually 1st visit, occasionally 2nd visit  □ Sometimes 1st visit, sometimes 2nd visit | | | | | | |
| **39. Date of visit** | □ Recorded  □ Not recorded ⇨ Skip | | □ Always 1st visit  □ Always 2nd visit | | | | | □ Usually 1st visit, occasionally 2nd visit  □ Sometimes 1st visit, sometimes 2nd visit | | | | | | |
| **40. Age** | □ Recorded  □ Not recorded ⇨ Skip | | □ Always 1st visit  □ Always 2nd visit | | | | | □ Usually 1st visit, occasionally 2nd visit  □ Sometimes 1st visit, sometimes 2nd visit | | | | | | |
| **41. Parity** | □ Recorded  □ Not recorded ⇨ Skip | | □ Always 1st visit  □ Always 2nd visit | | | | | □ Usually 1st visit, occasionally 2nd visit  □ Sometimes 1st visit, sometimes 2nd visit | | | | | | |
| **42. Gravidity** | □ Recorded  □ Not recorded ⇨ Skip | | □ Always 1st visit  □ Always 2nd visit | | | | | □ Usually 1st visit, occasionally 2nd visit  □ Sometimes 1st visit, sometimes 2nd visit | | | | | | |
| **43. Residence** | □ Recorded  □ Not recorded ⇨ Skip | | □ Always 1st visit  □ Always 2nd visit | | | | | □ Usually 1st visit, occasionally 2nd visit  □ Sometimes 1st visit, sometimes 2nd visit | | | | | | |
| **44. Occupation** | □ Recorded  □ Not recorded ⇨ Skip | | □ Always 1st visit  □ Always 2nd visit | | | | | □ Usually 1st visit, occasionally 2nd visit  □ Sometimes 1st visit, sometimes 2nd visit | | | | | | |
| **45. HIV test date** | □ Recorded  □ Not recorded ⇨ Skip | | □ Always 1st visit  □ Always 2nd visit | | | | | □ Usually 1st visit, occasionally 2nd visit  □ Sometimes 1st visit, sometimes 2nd visit | | | | | | |
| **46. HIV test result** | □ Recorded  □ Not recorded ⇨ Skip | | □ Always 1st visit  □ Always 2nd visit | | | | | □ Usually 1st visit, occasionally 2nd visit  □ Sometimes 1st visit, sometimes 2nd visit | | | | | | |
| **47. Syphilis test result** | □ Recorded  □ Not recorded ⇨ Skip | | □ Always 1st visit  □ Always 2nd visit | | | | | □ Usually 1st visit, occasionally 2nd visit  □ Sometimes 1st visit, sometimes 2nd visit | | | | | | |
| **48. On ART** | □ Recorded  □ Not recorded ⇨ Skip | | □ Always 1st visit  □ Always 2nd visit | | | | | □ Usually 1st visit, occasionally 2nd visit  □ Sometimes 1st visit, sometimes 2nd visit | | | | | | |
| 1. **Longitudinal Registers** | | | | | | | | | | | | | | |
| INSTRUCTIONS: This section asks about longitudinal registers: registers that are organized by mother (in which each line contains information about multiple visits by a single mother) as opposed to registers organized by date (in which each visit by a single mother is on a different line). Please explain the definition of longitudinal register to the interviewee. | | | | | | | | | | | | | | |
| **49. Is the ANC register a longitudinal register?** | | | | | □ No  □ Yes | | | | | | | | | |
| **50. Is the Laboratory testing register a longitudinal register?** | | | | | □ No  □ Yes | | | | | | | | | |
| 1. **Previously known positive women** | | | | | | | | | | | | | | |
| INSTRUCTIONS: This section collects information about pregnant women who already know they are HIV positive, what kind of PMTCT testing services they receive and how their information is recorded. | | | | | | | | | | | | | | |
| **51. If a pregnant woman already knows she is HIV positive, is she still given an HIV test for PMTCT?** | | | □ No ⇨  □ Yes | | | | **51a.** What is recorded in the women’s HIV test result field?  □ Positive  □ Known positive / on ARV  □ HIV Status at booking □ Nothing recorded  □ Other _______ | | | | | | | |
| **52. Is any information recorded to indicate that pregnant woman *already* knows she is HIV positive (to distinguish her from a women who has just been tested HIV positive by PMTCT services during the current pregnancy)?** | | | □ No  □ Yes ⇨ | | | | **52a.** If Yes to 52, in which column is this distinguishing information recorded?  □ HIV tested  □ HIV test result  □ Notes/comments  □ Other ________ | | | | | | | |
| 1. **Test refusal** | | | | | | | | | | | | | | |
| **53. If a woman who does NOT identify herself as already known to be HIV positive refuses an HIV test, what is recorded for “HIV test result”?** | | | | □ Not applicable  □ Refused  □ Nothing recorded  □ Other _______ | | | | | | | | | | |
| 1. **PMTCT monthly summary forms** | | | | | | | | | | | | | | |
| INSTRUCTIONS: Ask to see the PMTCT monthly summary forms from the ANC sentinel surveillance period Looking at the monthly summary forms for the period during the ANC sentinel surveillance period, record the following variables | | | | | | | | | | | | | | |
| **54. The number of women attending ANC for a new pregnancy** | | | | | | | | | | | | |  | |
| **55. The number of women who were tested for HIV through the PMTCT program, at their first visit** | | | | | | | | | | | | |  | |
| **56. The number of previously know positive pregnant women** | | | | | | | | | | | | |  | |
| **57. The number of newly identified positive pregnant women** | | | | | | | | | | | | |  | |
| 1. **Patient flow walk through** | | | | | | | | | | | | | | |
| Instructions: The goal of this section is to understand in more depth the flow of a woman through the clinic and the collection of her data. This will be done by physically walking through the entire process while each step is explained and recorded below. You may use information collected in sections 1-4 to probe or clarify responses. You may also use this section to further describe non-standard practices not adequately captured before now. Each time the interviewee indicates that a piece of information (e.g. age) is recorded in a certain place (e.g. ANC register) ask to look and visually verify that it is actually recorded there. Make sure that the walk through covers each piece of information that is collected (e.g., age, occupation, etc), where it is recorded (e.g., registers, etc), when it is recorded and how it is recorded. | | | | | | | | | | | | | | |
| **58. Please walk me through the entire process by which a pregnant woman moves through the clinic during her first ANC visit, from the time she enters to her departure. At every step of the way, please describe in detail when patient information is collected, where it is recorded and who is responsible for recording it.** | | | | | | | | | | | | | | |
| Instructions: The following questions should be asked only if this information was not described (or not described in enough detail) in Question 28. | | | | | | | | | | | | | | |
| **59. Are laboratory facilities for HIV testing at this site or at another location?**  (PROBE: If off-site, at what point of a woman’s first visit does she go to the lab? Does she get her HIV test results back the same day? If test results are not provided the same day, when do women receive their test results?) | | | | | | | | | | | | | | |
| **60. Please describe how the process and flow through the clinic differs for women who already know that they are HIV positive.**  (PROBE: Is this information recorded anywhere? If yes, ask to be shown where and how it is recorded. Are the women still tested for HIV? | | | | | | | | | | | | | | |
| **61. If a woman tests positive during this visit, how do the clinic procedures differ from those who test HIV negative?**  (PROBE: Are there different registers where information only for HIV positive pregnant women is recorded?) | | | | | | | | | | | | | | |
| **62. If a woman refuses an HIV test, what are the procedures? Is this information recorded? If yes, where is this recorded? Are reasons for refusing HIV testing documented?** | | | | | | | | | | | | | | |
| **63. Where are HIV test results recorded?**  (PROBE: Are HIV test results recorded in one or multiple documents? Where is this information recorded for the first time? Please ask to review the records to verify HIV testing data collection.) | | | | | | | | | | | | | | |
| INSTRUCTIONS: Thank the interviewee for his or her time and assistance. | | | | | | | | | | | | | | |
